# Insulin-like growth factor binding protein-1 regulates HIF-1α degradation to inhibit apoptosis in hypoxic cardiomyocytes

**DOI:** 10.1038/s41420-021-00629-3

**Published:** 2021-09-16

**Authors:** Xiaoyan Tang, Huilin Jiang, Peiyi Lin, Zhenhui Zhang, Meiting Chen, Yi Zhang, Junrong Mo, Yongcheng Zhu, Ningning Liu, Xiaohui Chen

**Affiliations:** 1 Department of Emergency, the Second Affiliated Hospital, Guangzhou Medical University, 510260 Guangzhou, Guangdong China; 2 Guangzhou Institute of Cardiovascular Disease, Guangdong Key Laboratory of Vascular Diseases, State Key Laboratory of Respiratory Disease, the Second Affiliated Hospital, Guangzhou Medical University, 510260 Guangzhou, China

**Keywords:** Myocardial infarction, Translational research

## Abstract

Hypoxia is important in ischemic heart disease. Excessive Insulin-like growth factor binding protein-1 (IGFBP-1) amounts are considered to harm cardiomyocytes in acute myocardial infarction. However, the mechanisms by which IGFBP-1 affects cardiomyocytes remain undefined. The present study demonstrated that hypoxia up-regulates IGFBP-1 and HIF-1α protein expression in cardiomyocytes. Subsequent assays showed that IGFBP-1 suppression decreased HIF-1α expression and inhibited hypoxia-induced apoptosis in cardiomyocytes, which was reversed by HIF-1α overexpression, indicating that HIF-1α is essential to IGFBP-1 function in cellular apoptosis. In addition, we showed that IGFBP-1 regulated HIF-1α stabilization through interacting with VHL. The present findings suggest that IGFBP-1–HIF-1α could be targeted for treating ischemic heart disease.

## Introduction

Acute myocardial infarction (AMI) represents the heart disease with the highest morbidity and mortality around the world [1]. It is widely accepted that apoptosis in cardiomyocytes (CMs) contributes to pathogenetic events in multiple cardiac diseases, including hypertrophic cardiomyopathy, ischemic heart disease, and congestive heart failure [2]. Meanwhile, the insulin-like growth factor (IGF) system is highly involved in the pathological mechanisms of cardiovascular diseases [3, 4].

The IGF system includes IGF-1 and IGF-2, type 1 and type 2 IGF receptors (IGF1R and IGF2R), and six binding proteins (IGFBPs). IGF bioavailability is rigorously controlled by six ubiquitous IGF-binding proteins found in the majority of tissue types. In circulation, IGFBPs interact with IGFs to suppress receptor activation and increase IGF’s half-life [5]. The IGF signaling pathway plays an important role in myocardial protection and heart development, such as IGFBP-3 [6, 7] and IGFBP-5 [8, 9]. IGFBP-1, one of the six IGFBP subtypes, has a molecular weight of about 30 kDa [10]. It is mainly produced in the liver, kidneys, and heart [11]. IGFBP-1 regulates cell proliferation, migration, and glucose metabolism by activating integrin-ILK/FAK/PTEN signaling through integrin receptors on the cell membrane [12–14]. Recently, I  GFBP-1 has attracted mounting attention as a biomarker in various cardiovascular events, such as atherosclerosis [15], AMI [16], and chronic heart failure [17]. Moreover, circulating IGFBP-1 amounts are markedly elevated in individuals with critical coronary artery disease (CAD) in comparison with those suffering from milder CAD [18]. Therefore, the mechanisms underpinning IGFBP-1’s effects are of great importance in the cardiovascular research field and could help develop novel therapeutic options for heart diseases. However, IGFBP-1 expression and molecular function in cardiovascular diseases remain elusive.

Cardiomyocyte stress is induced by hypoxic conditions immediately following the obstruction of blood flow in the coronary artery in AMI. Mounting evidence suggests that hypoxia triggers apoptotic events in CMs [19]. In addition, multiple cellular events induced by low-oxygen conditions are molecularly regulated by the transcriptional activities of hypoxia-inducible factors (HIFs) [20], with a significant contribution to oxygen homeostasis. HIF-1 exists as an α/β heterodimer, in which activation of the α-subunit depends on oxygen, while HIF-1β is constitutively expressed. Under normoxic conditions, HIF is hydroxylated by prolyl hydroxylases (PHDs), allowing HIF-1α to bind to the von Hippel–Lindau (VHL) protein and inducing its rapid degradation via the ubiquitin–proteasome pathway [21]. Hypoxia suppresses HIF-1α hydroxylation, resulting in protein stabilization and transfer to the nucleus. Then, HIF-1α and HIF-1β form a heterodimer, which interacts with hypoxia-responsive elements (HREs) in the promoter regions of more than 100 genes, thereby inducing the expression of downstream proteins, including vascular endothelial growth factor (VEGF) [22], inducible nitric oxide synthase (iNOS) [23] and GBP-1 [24, 25], and mediating the adaptive response to hypoxia. The downstream regulatory events promote essential adaptive processes such as glycolysis and angiogenesis while also driving anti-survival [26, 27] or pro-survival [28, 29] signaling in cancer and cardiac myocytes. Thus, identifying factors that regulate HIF-1α activity would provide valuable insights into the basic biological mechanisms and help develop improved therapeutic approaches.

We previously reported that IGFBP-1 amounts are elevated in the serum of patients with AMI as detected by the antibody array technology [30]. However, the mechanism and biological significance of IGFBP-1 in AMI remains unclear. In this study, we utilized neonatal rat ventricular CMs in conjunction with H9c2 cells as in vitro model, and the AMI model as in vivo model. The aim of this study was to establish the molecular role of IGFBP-1 in the HIF-1α pathway and hypoxia-induced apoptosis. We showed that IGFBP-1 protein expression has a positive correlation with HIF-1α protein levels in cardiac myocytes. Further investigation revealed that IGFBP-1 impacts HIF-1α protein stability via interaction with VHL. This feed-forward loop between IGFBP-1 and HIF-1α could suppress hypoxia-induced apoptosis in vitro and in vivo.

## Results

### IGFBP-1 is upregulated in hypoxic condition

**Fig. 1:**
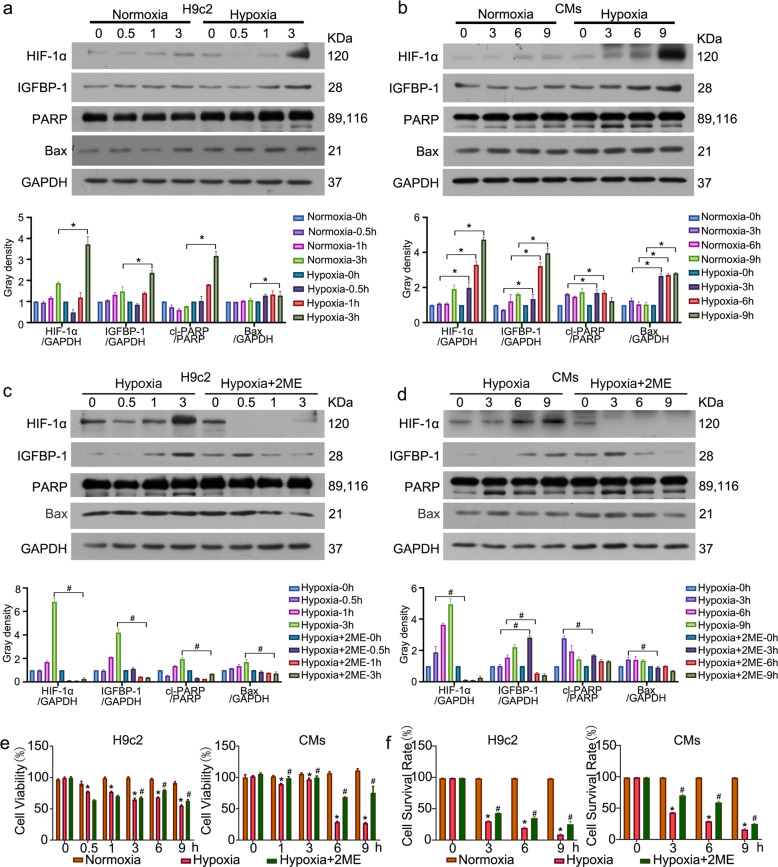
Hypoxia upregulates IGFBP-1 expression. H9c2 cells and CMs were incubated under normoxic and hypoxic conditions for various times. **a**, **b** Western blot and quantification was performed to measure the protein levels of IGFBP-1, HIF-1α, PARP, and Bax under normoxic and hypoxic conditions. GAPDH was served as a loading control. **c**, **d** Following treatment with 2-ME (100 μM) for the indicated times, the protein amounts of IGFBP-1, HIF-1α, PARP, and Bax were measured by immunoblot. GAPDH was served as a loading control. **e** Cell viability was detected by the MTS assay. **f** Apoptotic cells were detected with Annexin V-APC/7AAD followed by flow cytometry analysis. Cell survival rate was shown. Data were presented as mean ± standard deviation (SD). ^*^P < 0.05 vs. the normoxia group at the same time point, ^#^*P* < 0.05 vs.^.^ the hypoxia group at the same time point. two-way ANOVA was performed. Three independent experiments were performed.

To decipher IGFBP-1 function under pathological conditions, including hypoxia, in CMs and H9c2 cells, we analyzed IGFBP-1 and HIF-1α protein amounts under hypoxic conditions. As shown in Fig. 1a and b, IGFBP-1 and HIF-1α were upregulated in hypoxia, which was accompanied by elevated amounts of the apoptotic proteins PARP and Bax. To further verify HIF-1α involvement in hypoxia-related IGFBP-1 upregulation, we adopted a pharmacological approach (treatment with 2-methoxyestradiol, 2-ME) to inhibit HIF-1α. Fig. 1c and d showed notably decreased IGFBP-1 and HIF-1α protein amounts after treatment with 2-ME. PARP and Bax amounts were also reduced by HIF-1α inhibition. All the results showed that HIF-1α inhibition leads to decreased IGFBP-1 protein levels. The MTS assay and flow cytometry analysis was performed to further confirm that cell viability was decreased under hypoxic conditions, and HIF-1α inhibition reversed the suppressed cell viability (Fig. 1e) and apoptosis (Fig. 1f and Supplementary Fig. 1) of H9c2 cells and CMs in hypoxia. As prolonged hypoxia, the cell survival rate of hypoxia for 9 h is <20%, and the cytoprotective effect caused by inhibition of HIF-1α is weakened. Therefore, we choose 3 and 6 h as the time points for the further experiments. Jointly, the above data revealed IGFBP-1 as a hypoxia-regulated protein in H9c2 cells and CMs, and inhibiting HIF-1α has a protective effect on cell apoptosis during the acute hypoxia phase.

### Knockdown of IGFBP-1 inhibits hypoxia-induced apoptosis and depends on HIF-1α status

**Fig. 2:**
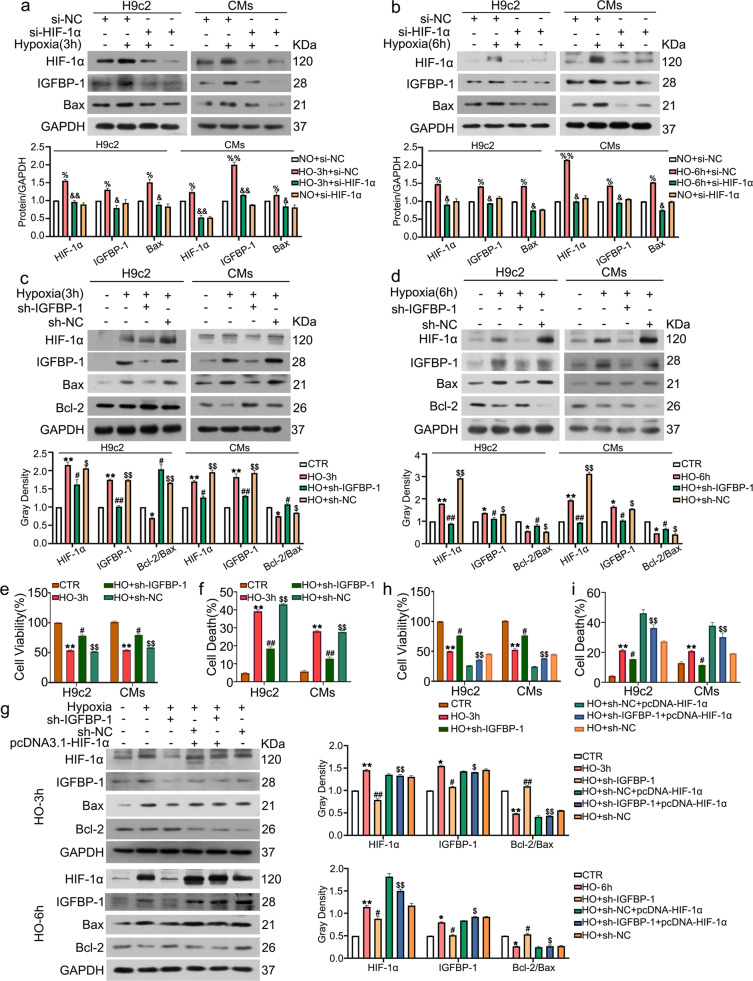
Knockdown of IGFBP-1 inhibits cell apoptosis and depends on HIF-1α status. H9c2 cells and CMs were transfected with HIF-1α siRNA and then exposed to hypoxia for 3 h (**a**) and 6 h (**b**). Protein lysates were prepared for western blotting for HIF-1α, IGFBP-1 and Bax. GAPDH was served as a loading control. Quantitative data of band gray density are shown. H9c2 cells and CMs were infected with Ad- shIGFBP-1 (MOI of 10) or negative control for 48 h and exposed to hypoxia for 3 h (**c**) and 6 h (**d**). Immunoblot was carried out to assess HIF-1α, IGFBP-1, Bax, and Bcl-2 protein amounts. GAPDH was served as a loading control. Quantitative data of band gray density are shown. **e** A total of 20 μl of MTS reagent was added for 2 h, and cell viability was measured. **f** Cell death population of H9c2 cells and CMs were shown from the three independent replicates. H9c2 cells were co-transfected with the pcDNA3. 1-HIF-1α plasmid and Ad-shIGFBP-1 for 48 h and exposed to hypoxia for 3 h (upper 5 lanes of **g**) and 6 h (lower 5 lanes of **g**). Immunoblot and quantification were carried out to assess HIF-1α, IGFBP-1, Bax, and Bcl-2 protein amounts. GAPDH was served as a loading control. **h** Cell viability of H9c2 cells and CMs were determined after incubation with the MTS reagent for 2 h. **i** Cell death population of H9c2 cells and CMs were shown from the three independent replicates. Data were presented as mean ± standard deviation (SD). ^%^*P* < 0.05, ^%%^*P* < 0.01 vs. normoxia + si-NC group; ^&^*P* < 0.05, ^&&^*P* < 0.01 vs. hypoxia + si-NC group; ^*^P < 0.05, ^**^P < 0.01 vs. CTR group; ^#^*P* < 0.05, ^##^*P* < 0.01 vs. indicating HO group; ^$^*P* < 0.05, ^$$^*P* < 0.01 vs. HO + sh-IGFBP-1 group, one-way ANOVA followed by SNK multiple comparison test performed. Three independent experiments were performed.

To exclude the non-specificity of 2-ME, a siRNA of HIF-1α was used to transfect H9c2 cells and CMs, followed by determining effects on the level of IGFBP-1 and Bax protein. Similarly, HIF-1α knockdown induced a dramatic reduction of IGFBP-1 and Bax in 3 h (Fig. 2a) and 6 h (Fig. 2b). As prolonged hypoxia in 9 h (Supplementary Fig. 2a), compared with hypoxia + siNC group, si-HIF-1α decreased the protein expression of HIF-1α and IGFBP-1 in hypoxia + si-HIF-1 group, however, the expression of Bax did not change. The results showed that HIF-1α inhibition leads to decreased IGFBP-1 protein levels.

Due to the upregulation of IGFBP-1 in hypoxia, we further explored whether IGFBP-1 is important in hypoxia-induced apoptosis. IGFBP-1 was silenced in both H9c2 cells and CMs under hypoxic conditions, before apoptosis evaluation. To evaluate the functional importance of IGFBP-1 to HIF-1α, IGFBP-1’s effect on HIF-1α expression was determined. Fig. 2c and d showed that the knockdown of IGFBP-1 dramatically decreased HIF-1α protein amounts in hypoxia for 3 and 6 h. Similar to HIF-1α decrease, classical pro-apoptotic protein Bax were downregulated after IGFBP-1 silencing, while Bcl-2 (an anti-apoptotic protein) amounts were increased. However, the expression of Bax and Bcl-2 did not change in 9 h (Supplementary Fig. 2b). HIF-1α target genes, including BNIP-3 and p53, were also downregulated in mRNA level (Supplementary Fig. 2c). As shown in Fig. 2e, decreased cell viability in H9c2 cells and CMs under hypoxic conditions was alleviated by Ad-shIGFBP-1 silencing. Consistent with MTS assay, flow cytometry analysis showed that knockdown of IGFBP-1 potently blocked hypoxia-induced cell death (Fig. 2f and Supplementary Fig. 2d). These results indicated that knockdown of IGFBP-1 led to decreased HIF-1α protein levels and protected CMs from apoptosis in hypoxia.

Given that IGFBP-1 regulates hypoxia-induced apoptosis and HIF-1α protein expression, we next assessed whether IGFBP-1-related HIF-1α inhibition is crucial for rescuing cell apoptosis. To this end, we transfected the pcDNA3.1 plasmid overexpressing HIF-1α into H9c2 cells and assessed cell apoptosis after IGFBP-1 knockdown in hypoxic conditions. As shown in Fig. 2g, downregulation of HIF-1α, IGFBP-1, Bax protein level, and upregulation of Bcl-2 induced by IGFBP-1 deletion were reversed upon HIF-1α overexpression in hypoxia for 3 and 6 h. However, the expression of Bax and Bcl-2 did not change in 9 h (Supplementary Fig. 2e). In Fig. 2h, the MTS assay showed HIF-1α overexpression potently inhibited cell viability increase induced by IGFBP-1 knockdown. Flow cytometry analysis further confirmed that HIF-1α overexpression resulted in enhanced hypoxia-induced apoptosis and suppressed the protection afforded by IGFBP-1 knockdown (Fig. 2i and Supplementary Fig. 2f). Taken together, these results indicated that IGFBP-1-associated cell apoptosis inhibition depends on HIF-1α status.

### IGFBP-1 regulates HIF-1α stability

**Fig. 3:**
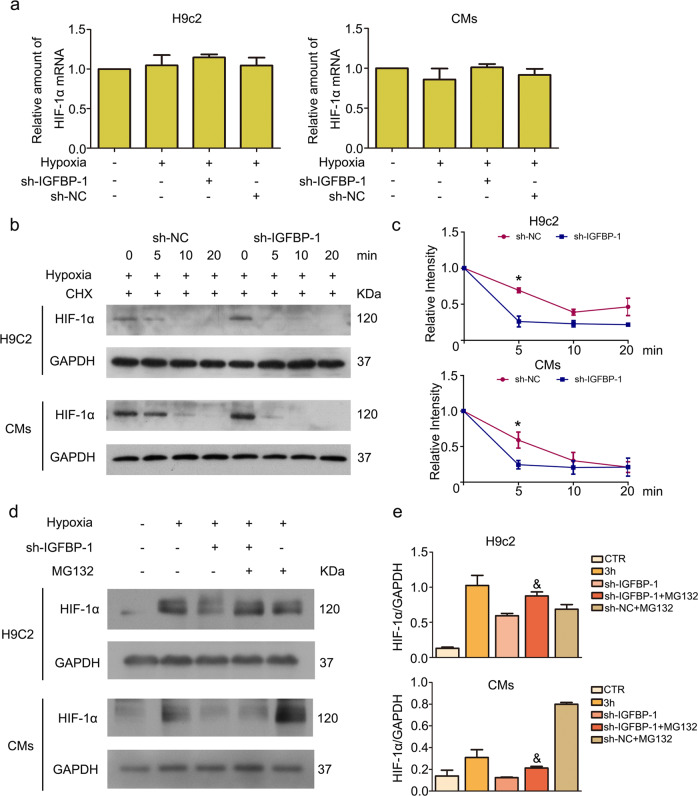
Knockdown of IGFBP-1 decreases HIF-1α stability. **a** H9c2 cells and CMs underwent infection withAd-shIGFBP-1 (MOI of 10) or negative control for 48 h. Total RNA was obtained and assessed by qRT-PCR for HIF-1α mRNA amounts. *p* > 0.05 versus control. **b** H9c2 cells and CMs infected with Ad-shIGFBP-1 and underwent incubation with CHX (50 μg/ml) in hypoxia for the indicate times. HIF-1α protein amounts were assessed by immunoblot. **d** H9c2 cells and CMs were infected with Ad-shIGFBP-1 and incubated with MG132 in hypoxia for 3 h. Western blot analysis was performed for detecting HIF-1α protein amounts. Quantitative data of HIF-1α (**c**, **e**) are shown. Data were presented as mean ± standard deviation (SD). ^*^P < 0.05 vs.^.^ sh-IGFBP-1 group, two-way ANOVA was performed. ^&^*P* < 0.05 vs. sh-IGFBP^-^1 group, one-way ANOVA followed by SNK multiple comparison test performed. Three independent experiments were performed.

Cellular levels of the HIF-1α protein are regulated via transcription, translation, and protein degradation. Here, we first determined whether knockdown of IGFBP-1 inhibits HIF-1α gene transcription in H9c2 cells and CMs under hypoxic conditions for 3 h. In this study, qRT-PCR demonstrated that IGFBP-1 knockdown had negligible effects on HIF-1α gene transcription (Fig. 3a). To further assess the potential regulatory function of IGFBP-1 on HIF-1α, we assessed HIF-1α degradation after sh-NC and sh-IGFBP-1 treatment in hypoxia with CHX. Immunoblot suggested that the reduced half-life of HIF-1α was overtly correlated with IGFBP-1 knockdown (Fig. 3b and c). As HIF-1α undergoes oxygen-dependent hydroxylation and ubiquitination in normoxic conditions, promoting its degradation by the proteasome system, we hypothesized that sh-IGFBP-1promotes HIF-1α degradation via the proteasome system. Notably, HIF-1α protein level reduction following IGFBP-1 silencing was alleviated by the proteasome inhibitor MG132 (Fig. 3d and e), suggesting that IGFBP-1 is functionally essential for HIF-1α stability.

### IGFBP-1 suppresses ubiquitination of HIF-1α via interaction with VHL

**Fig. 4:**
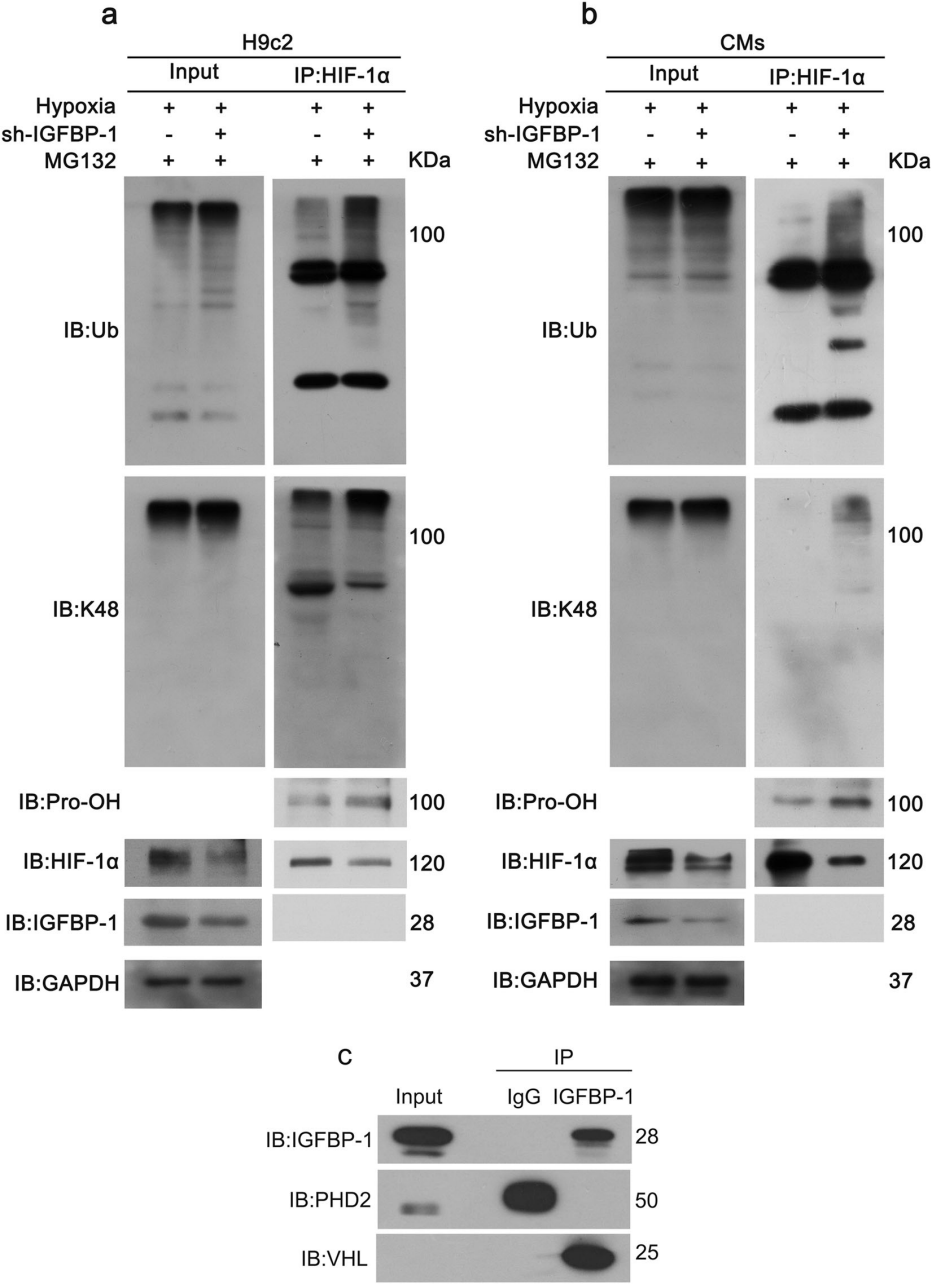
IGFBP-1 regulates HIF-1 αubiquitination by interacts with VHL. **a**, **b** H9c2 cells and CMs underwent infection with Ad-shIGFBP-1 (MOI of 10) and incubated with MG132 (10 μM), followed by a 3 h exposure to hypoxia. Cells were harvested for immunoprecipitation with HIF-1α and assessed by immunoblot for ubiquitin (Ub), lys48 ubiquitin (K48), hydroxyproline (Pro-OH) and IGFBP-1. **c** H9c2 cells were transfected with pcDNA3.1-HIF-1α plasmids and IGFBP-1 adenovirus for 48 h, followed by IGFBP-1 IP and western blotting for PHD2 and VHL. Three independent experiments were performed.

Proteins are in a dynamic equilibrium in vivo, with continuous synthesis and degradation throughout life [31], we hypothesized that the HIF-1α downregulation triggered by IGFBP-1 knockdown results from HIF-1α degradation. To test this hypothesis, we next investigated whether IGFBP-1 silencing affects HIF-1α ubiquitination. A total of 48 h after Ad-shIGFBP-1 infection, cells underwent culture under hypoxic conditions in presence of MG132 (10 μM) for 3 h. Cell lysates underwent immunoprecipitation with anti-HIF-1α antibodies and immunoblot for ubiquitin, lys48 ubiquitin (K48), hydroxyproline (Pro-OH), and IGFBP-1 detection. We found that IGFBP-1 knockdown dramatically increased the expression of HIF-1α hydroxylation and ubiquitination, both in H9c2 cells (Fig. 4a) and CMs (Fig. 4b). These findings suggested that IGFBP-1’s effects on HIF-1α stabilization occurs via suppression of its hydroxylation and ubiquitination.

It is well known that HIF-1α is hydroxylated by PHDs under normoxic conditions, then binds to the VHL protein and inducing its rapid degradation via the ubiquitin–proteasome pathway. In order to find out the direct binding protein of IGFBP-1, we overexpressed HIF-1α and IGFBP-1 under normoxia conditions. Cell lysates were immunoprecipitated with IGFBP-1 antibodies, followed by immunoblot with antibodies against PHD2 and VHL proteins, which directly affected HIF-1α hydroxylation and ubiquitination. As shown in Fig. 4c, no association at all of IGFBP-1 and HIF-1α. IGFBP-1 mainly binds to VHL, then blocks the ubiquitination of HIF-1α, thereby inhibiting the degradation. Moreover, we have overexpressed the same levels of HIF-1α and different levels of IGFBP-1 under normoxia conditions, and co-immunoprecipitation assays using anti-IGFBP-1 and anti-VHL antibodies have been performed. As shown in Supplementary Fig. 3a, IGFBP-1 dose-dependently increased the interaction of VHL and IGFBP-1, which can increase the stability of HIF-1α. Furthermore, co-immunoprecipitation assays using VHL and anti-HIF-1α showed that less HIF-1α and VHL association in different overexpression levels of IGFBP-1 in H9c2 cells (Supplementary Fig. 3b). Taken together, these findings strongly suggested that IGFBP-1 regulates HIF-1α stability through the association with VHL, not by its direct binding to HIF-1α.

### Knockdown of IGFBP-1 ameliorates myocardial injury after AMI

**Fig. 5:**
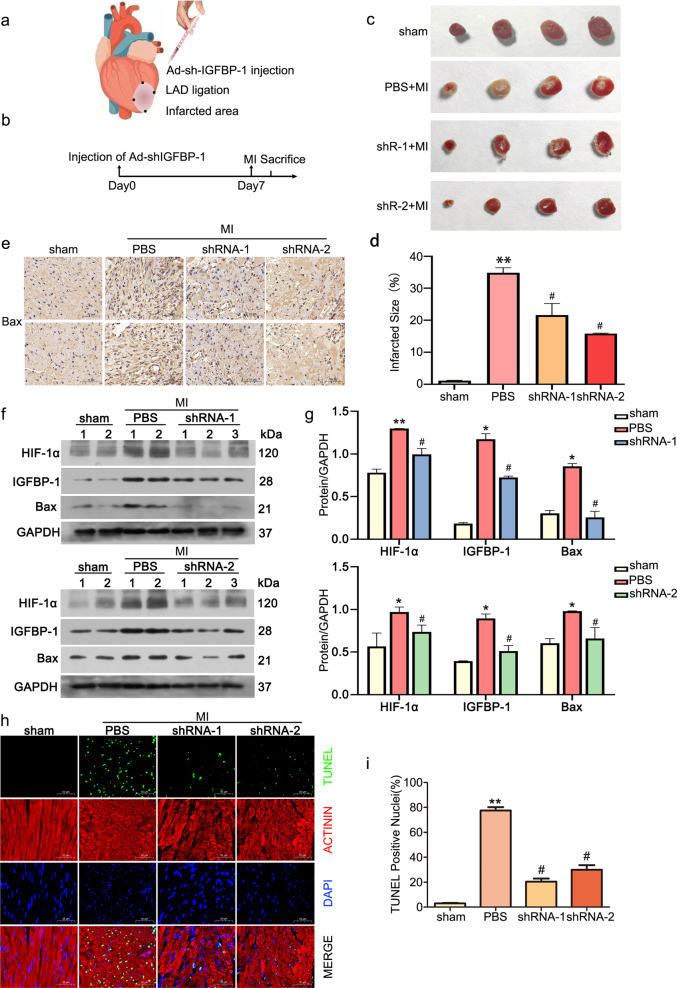
IGFBP-1 inhibition suppresses cardiomyocyte apoptosis in mice with myocardial infarction. **a** Schematic representation of myocardial infarction modeling and intramyocardial injection of Ad-shIGFBP-1in vivo. **b** Schematic depiction of Ad-shIGFBP-1 injection and sample collection. **c**, **d** Examination of myocardial infarct size in mice with myocardial infarction by TTC staining. Red indicates nonnecrotic myocardium; white indicates necrotic myocardium. **e** The Bax protein within the ischemic heart tissue was analyzed immunohistochemically (×200). **f** Immunoblot assessed HIF-1α, IGFBP-1, and Bax protein amounts in ischemic heart tissue at 6 h after LAD ligation, normalized by GAPDH. **g** Quantitative data of HIF-1α, IGFBP-1 and Bax are shown, respectively. **h** Apoptosis of cardiac myocytes at 6 h in ischemic myocardium was determined by TUNEL. TUNEL-positive cells are green. Cadiomyocytes are stained with actinin (red). Nuclei are counterstained with DAPI(blue). Scale Bar = 50 μm. **i** Quantitation of cardiac myocytes apoptosis. All data were presented as mean ± standard deviation (SD). ^*^P < 0.05, ^**^P < 0.01 vs^.^ sham group; ^#^*P* < 0.05 vs. PBS group^,^ one-way ANOVA followed by SNK multiple comparison tests performed. *N* = 12 mice for each group.

In the previous part, we confirmed that under hypoxic conditions, IGFBP-1 affects myocardial cell apoptosis by regulating the stabilization of HIF-1α. In this part, we will further explore the protective effect of sh-IGFBP-1 in AMI model. Seven days after injection of the adenovirus-shRNA-IGFBP-1, the animal hearts were subjected to LAD ligation (Fig. 5a). Six hours later, the mice were euthanized for sample collection (Fig. 5b). After AMI, apoptosis of CMs occurred alongside ischemia and hypoxia.

In order to understand the effect of sh-IGFBP-1 on myocardial infarction, we performed TTC staining to observe the infarct size. In Fig. 5c and d, the result showed that compared with PBS, sh-IGFBP-1 could reduce infarct size after AMI. Immunohistochemical staining confirmed that IGFBP-1 silencing protected the heart tissue by reducing the protein amounts of Bax upon ischemia (Fig. 5e). The western blotting analysis further confirmed that adenovirus-shRNA-IGFBP-1 injection resulted in decreased HIF-1α and IGFBP-1 protein amounts in the ischemic myocardium. As IGFBP-1 decreased, the protein amounts of Bax in ischemic heart tissue also decreased (Fig. 5f and g). In the TUNEL assay, TUNEL-positive (green) apoptotic cardiac myocytes in the shRNA-1 and shRNA-2 groups were reduced in comparison with the PBS group (Fig. 5h and i). Taken together, compared with PBS, IGFBP-1 silencing can reduce infarct size and attenuate apoptosis in CMs, and relieved myocardial damage upon AMI.

### IGFBP-1 depletion has a protective effect on cardiac function and reduces cardiac fibrosis 28 days after MI

**Fig. 6:**
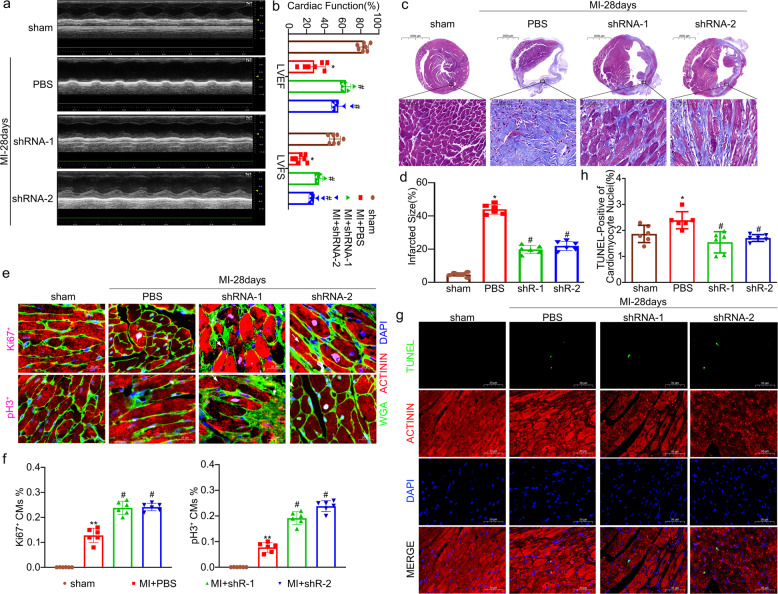
IGFBP-1 depletion increased functional recovery and myocardial repair 28 days after MI in mice. Mice were maintained for 28 days after LAD ligation. **a**, **b** Cardiac function was assessed by ultrasound examination, then used to calculate the left ventricular ejection fraction (LVEF) and fractional shortening (LVFS). **c** Masson staining revealed more preserved myocardium and decreased interstitial fibrosis in the infarct zone (28 days post-MI) of IGFBP-1 depletion mice. Scale bar = 2000 and 50 μm. **d** Quantitation of collagen volume fraction in the infarct zone. **e** Representative Immunofluorescence staining for Ki67 and pH3. Scale bar = 20 μm. **f** Quantitative analysis of Ki67^+^cardiomyocytes and pH3^+^ cardiomyocytes in the border zone 28 days after MI. **g** Apoptosis of cardiac myocytes at 28 days was determined by TUNEL. TUNEL-positive cells are green. Cadiomyocytes are stained with actinin (red). Nuclei are counterstained with DAPI (blue). Scale Bar = 50 μm.**h** Quantitation of cardiac myocytes apoptosis. All data were presented as mean ± standard deviation (SD). ^*^P < 0.05 vs. sham group; ^#^*P* < 0.05 vs.^.^ *P*BS group, one-way ANOVA followed by SNK multiple comparison test performed. *N* = 6 mice for each group.

In order to find out whether knockdown of IGFBP-1 has a potential therapeutic effect on myocardial infarction, we maintained injected animals for 28 days after LAD ligation and assessed cardiac function by ultrasound examination. As we have shown in Fig. 6a and b, LVEF and LVFS of the shRNA-1 group and shRNA-2 groups were increased in comparison with the PBS group. In addition, we assessed the fibrosis in the infarcted heart by Masson staining. The results of the Masson staining showed that (Fig. 6c and d), compared with the PBS group, a more preserved myocardium and decreased interstitial fibrosis in the infarct zone of the shRNA-1 group and shRNA-2 groups. In addition, we performed immunofluorescence staining of nuclear division markers (Ki67, pH3) to observe the proliferation of CMs. As shown in Fig. 6e and f, an increased number of Ki67^+^ CMs and pH3^+^ CMs in the border zone of the shRNA-1 group and shRNA-2 group in comparison to the PBS group (Fig. 6e and f). In the TUNEL assay, myocardial apoptosis is <7 days after myocardial infarction, and TUNEL-positive (green) apoptotic CMs in the shRNA-1 and shRNA-2 groups were reduced in comparison with the PBS group at 28 days after MI (Fig. 6g and h). These findings suggest that knocking down IGFBP-1 has a protective effect on cardiac function and reduces cardiac fibrosis 28 days after MI.

## Discussion

The role of IGFBP-1 as a transporter of IGF-1, and its RGD region, in activating integrin receptors in various cancer cells is well-known [32, 33]. However, current research is mainly focused on cancers, and its potential function in CMs under pathological conditions, e.g. hypoxia, is rarely investigated. This study identifies IGFBP-1 as a hypoxia-regulated molecule directly promoting HIF-1α protein stabilization in heart-derived H9c2 cells and neonatal rat CMs, thereby controlling hypoxia-induced cell apoptosis.

It is well documented that hypoxia causes multiple molecular alterations with rapid and substantial physiological consequences in cells, and HIF-1α contributes to apoptosis and cytokine regulation [22, 34]. HIF-1, comprising the HIF-1α and HIF-1β subunits, induces hypoxia-sensitive genes by interacting with the HREs at functionally critical binding sites [35]. It is essential for the cellular response to hypoxia as it serves to transactivate a number of genes responsible for cellular survival. Besides, HIF-1 stimulates the intrinsic apoptotic pathway and cell death in hypoxia [29, 36]. Previous reports have noted thatIGFBP-1 gene expression and protein amounts time-dependently change in hypoxia, likely involving HIF-1α activation [12, 37, 38]. Our study demonstrates that, hypoxia time-dependently promoted IGFBP-1 and HIF-1α upregulation in cardiac myocytes. Furthermore, hypoxia-induced IGFBP-1 protein expression and cell apoptosis were obviously blocked by the HIF-1α specific inhibitor 2-ME and siRNAin H9c2 cells and CMs, further indicating the important function of HIF-1α in hypoxia-related IGFBP-1 upregulation and cell apoptosis in CMs (Fig. 1). These data corroborate previous reports demonstrating that HIF-1α induces apoptosis in CMs and other cells [39–41]. Importantly, the interplay of HIF-1α-IGFBP-1 signaling modulated hypoxia-induced apoptosis in H9c2 cells and CMs, further revealing a significant function for IGFBP-1 as a hypoxia-regulated protein.

We further explored the molecular mechanism of IGFBP-1 in regulating cardiomyocyte apoptosis. Given the upregulation of IGFBP-1 in hypoxia, we constructed a knockdown tool, adenovirus-shRNA targeting IGFBP-1, to infect CMs. Here, we firstly found that IGFBP-1 was identified as a positive regulator of HIF-1α under hypoxic conditions. Furthermore, IGFBP-1 knockdown significantly suppressed hypoxia-induced apoptosis in both H9c2 cells and CMs. In particular, we found the protective effect of IGFBP-1 knockdown on CMs can be reversed by HIF-1α overexpression (Fig. 2). These findings suggeste that HIF-1α plays an important role in CMs protection induced by IGFBP-1 deletion.

As HIF-1α protein amounts primarily affected by the rate of protein degradation [42], we further explored the mechanism by which IGFBP-1 regulates HIF-1α and initially considered degradation as the most likely target ofAd-shIGFBP-1.As demonstrates in Fig. 3, MG132 partially blunted HIF-1α downregulation after the knockdown of IGFBP-1. In order to clarify the molecular mechanism by which IGFBP-1 regulates HIF degradation, we performed IP with H9c2 cells and CMs and the results showed IGFBP-1 had strong effects in regulating HIF-1α protein stability by reducing its proteasome-dependent degradation. Because HIF-1α is unstable under normoxic conditions, we further overexpressed HIF-1α and IGFBP-1 in H9c2 cells in normoxic conditions, immunoprecipitation was performed with IGFBP-1 antibodies, followed by immunoblot with antibodies against PHD2 and VHL proteins. Our study demonstrates IGFBP-1 regulates HIF-1α stability by binding with VHL, reducing the ubiquitination of HIF-1α, not by its direct binding to HIF-1α (Fig. 4). These findings are aligned with previous studies, such as STAT3 increases HIF-1α stability by inhibiting pVHL interaction with HIF-1α and reducing ubiquitination [43]. In addition, Ras association domain family 1A (RASSF1A) interacts with HIF-1α, reduces hydroxylation and proteasome-dependent degradation, and increases its transcriptional activity [44]. According to our results, IGFBP-1 binds to VHL and regulates the stability of HIF-1α through the above-mentioned mechanism.

**Fig. 7:**
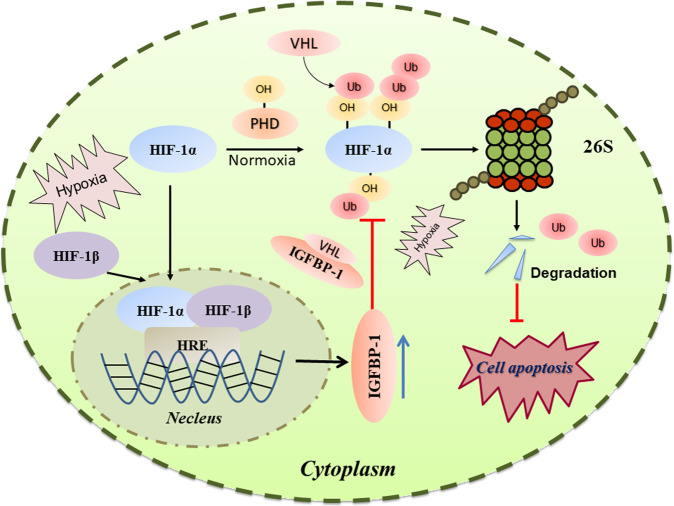
Schematic representation of IGFBP-1-mediated HIF-1α regulation. Our present finding demonstrated therole of IGFBP-1 in hypoxic cardiomyocytes. IGFBP-1 expression is increased in hypoxic condition and AMI model. IGFBP-1 interacts with VHL and suppresses HIF-1α degradation by ubiquitination. IGFBP-1 loss inhibits hypoxia-induced apoptosis in CMs and the AMI model via downregulating HIF-1α expression.

Apoptosis of CMs represents the primary reason for cardiomyocyte loss, which is related to cardiac remodeling in multiple cardiovascular pathologies. Several studies have suggested that IGFBP-1 constitutes an effective marker for assessing cardiovascular risk factors [45, 46], while its effects in the heart and the underpinning mechanisms have not been resolved. In the present study, we demonstrate that suppressing IGFBP-1 can protect the cardiomyocyte of AMI (Fig. 5). Moreover, the heart mainly manifests as myocardial fibrosis after 28 days after myocardial infarction, and knocking down of IGFBP-1 has a certain therapeutic effect on cardiac function and reduces cardiac fibrosis after 28 days of myocardial infarction (Fig. 6). In this study, we demonstrate that knockdown of IGFBP-1 could beneficially reduce apoptosis in cardiac myocytes of the infarct area upon acute MI, mainly by preventing HIF-1α ubiquitination (Fig. 7).

In conclusion, we firstly demonstrate a critical role for IGFBP-1 in regulating HIF-1α ubiquitination to hypoxia-induced apoptosis in CMs and the AMI model. The underpinning mechanisms describes in this study provide novel insights into therapeutic approaches, which can be employed to improve the treatment of hypoxia-induced heart cell apoptosis.

## Materials and methods

### Mice

C57BL/6 mice (20–23 g) were provided by the Guangdong Laboratory Animal Monitoring Institute and housed with freely available standard rodent chow and water. All experiments involving animals had approval from the Institutional Animal Care and Use Committee of Guangzhou Medical University (Guangzhou, China).

### Cell culture and hypoxia induction

Cardiac myocytes were obtained from the ventricles of Sprague–Dawley (SD) rats (1–2 days old) as described previously [47]. Briefly, left ventricles underwent collagenase Type II (Gibco, USA) digestion, with the resulting cells resuspended in DMEM supplemented with 10% fetal bovine serum (FBS). CMs were enriched by culture with 10% FBS-supplemented DMEM with 0.1 mM bromodeoxyuridine (BrdU). H9c2 cells provided by the American Type Culture Collection (ATCC, Manassas, VA, USA)underwent culture in DMEM containing 100 μg/mL penicillin, 100 IU/ml streptomycin, 2 mM glutamine (Gibco), and 10% FBS. To mimic ischemic damage in vitro, cells underwent exposure to glucose-free DMEM in a humidified hypoxic incubator with 94% N_2_, 5% CO_2,_ and 1% O_2_ for the indicated times. The control group was incubated under normoxic conditions (21% O_2_ and 5% CO_2_) at 37 °C. When indicated, the HIF-1α inhibitor 2-methoxyestradiol (2-ME; Selleck, USA) was added 1 h before hypoxia treatment.

### Reagents and antibodies

2-ME (S1233) and MG132 (S2619) were provided by Selleck. Cycloheximide (CHX) was provided by MedChemExpress (MCE, USA). MTS (G111) was a product of Promega (USA). The Co-IP assay kit (14311D) was provided by Life Technologies (USA). The Annexin V-FITC/PI apoptosis detection kit (KGA107) was provided by Keygen (China). Anti-PARP (9532), anti-Bax (14796), anti-Ubiquitin (3936), and anti-K48-ub (12805) antibodies were manufactured by Cell Signaling (USA). Anti-HIF-1α (ab179483) and anti-IGFBP-1 (ab181141) were from Abcam (UK). Anti-Bcl-2 (26593) was from Proteintech (USA). Anti-GAPDH (MB001) was provided by Bioworld Technology (USA). Immunoblot was performed with the antibodies diluted at 1:1000; for Co-IP, anti-HIF-1α antibodies (ab1) were utilized at 1:50. Anti-Bax antibodies for immunohistochemistry (Cell signaling) were utilized at 1:50. The HIF-1α siRNA (sc-45919) was obtained from Santa Cruz Biotechnology (Santa Cruz, CA, USA). The adenovirus and plasmids were provided by Vigene Biosciences (China) and GenePharma (China), respectively.

### Plasmid transfection and adenovirus infection

H9c2 cells underwent transient transfection with the pcDNA3.1-HIF-1α plasmid employing Lipofectamine 3000 (Invitrogen, USA) as directed by the manufacturer.

Adenovirus harboring shIGFBP-1 (Ad-shIGFBP-1) and negative control (sh-NC) were provided by Vigene Biosciences. Cell infection with Ad-shIGFBP-1 and sh-NC (multiplicity of infection [MOI] = 10), respectively, was performed for 48 h before exposure to hypoxic conditions.

### Cell viability assay

Cells seeded into 96-well plates in triplicate underwent overnight culture and infection with Ad-shIGFBP-1 or sh-NC for 48 h. Then, they were exposed to hypoxic conditions for 3 h, followed by the MTS assay, as previous report [48].

### SiRNA transfection assay

Cells were plated in dishes for 24 h. For siRNA transfection, the complex, including 500 μl RPMI opti-MEM, HIF-1α siRNA, and lipofectamine 3000 reagent was prepared to incubate with cells. After 6 h, fresh medium was replaced. Cells were collected after incubation for 48 h.

### Immunoblot

Immunoblot was carried out as described in a previous report [49]. In brief, protein samples were resolved by 12% SDS–PAGE and electro-transferred onto polyvinylidene difluoride (PVDF) membranes. After blocking with 5% skimmed milk in TBST (1 h at ambient), the membranes underwent successive incubations with primary (overnight at 4 °C) and HRP-linked secondary (1 h at ambient) antibodies. The ECL system (Santa Cruz) was employed for detection.

### Quantitative real-time polymerase chain reaction (qRT-PCR)

RNA extraction utilized TRIzol Reagent (Invitrogen). Reverse transcription was performed with 1 µg total RNA using the PrimeScript RT Master Mix kit (TaKaRa, Japan). HIF-1α and GAPDH mRNA amounts were assessed by qRT-PCR with the SYBR Premix Ex Taq kit, as directed by the manufacturer. The following primers were employed: HIF-1α: F: 5′-ATTCACAGCTCCCCAGCATT-3′, R: 5′-TAAGGGACAAACTCCCTCACC-3′; GAPDH: F:5′‘-TCACAATTCCATCCCAGACCC-3′, R: 5′-ATGGTATTCGAGAGAAGGGAGG-3′.

### Cell apoptosis assessment

Cell apoptosis was assessed with Annexin V-APC/7-AAD Apoptosis Kit (MultiSciences), as directed by the manufacturer. In brief, after resuspension in 200 μl binding buffer, the cells underwent incubation with 10 μl Annexin V-APC and 5 μl 7-AAD at ambient for 10 min. BD FACSVerse (BD Biosciences) flow cytometer was employed for data analysis.

### Co-immunoprecipitation (Co-IP)

Co-immunoprecipitation was performed as previously described [50]. In brief, Dynabeads and antibodies were mixed and incubated overnight, followed by further incubation with cell lysates for 1 h at 4 °C. Then, the Dynabeads were thrown off and an SDS loading buffer was applied to dissolve the mixtures. Finally, lysis was performed before assessment by immunoblot.

### In vivo adenoviral gene delivery

For MI model establishment, male mice (20–23 g) were anesthetized with isoflurane by inhalation, and tracheal intubation was performed via the oral cavity. Then, the animals were submitted to mechanical ventilation with an ALCBIO-V8S rodent ventilator. Left-side thoracotomy was carried out for heart exposure, and intramyocardial injection of 100 μl of diluted adenovirus (1 × 10^11^ PFU/ml) or sterile saline (100 μl) using a 30 G needle was performed. The adenovirus was injected at four sites into the apex of the heart, where the left ventricle is located. One week after intramyocardial injection, the surviving animals underwent re-thoracotomy, with the left anterior descending artery (LAD) ligated 2 mm from its origin between the pulmonary artery conus and the left atrium with 9-0 polyester sutures. Left ventricular muscle blanching and ST elevation by electrocardiography confirmed successful modeling.

### Immunohistochemical staining

Mouse euthanasia occurred 6 h after myocardial infarction. Fresh heart samples underwent fixation with 4% (w/v) paraformaldehyde, followed by paraffin embedding and sectioning at 5 μm. Then, immunohistochemistry was carried out as reported previously [51].

### Terminal deoxynucleotidyl transferase dUTP nick-end labeling (TUNEL) assay

6 h following modeling and treatments, apoptosis in cardiac myocytes in infarcted heart tissues was assessed with TUNEL Assay Kit (Roche) as directed by the manufacturer, under a confocal microscope.

### Determination of myocardial infarct size in rats with AMI

At the end of the experiment, the heart was rapidly excised, washed with 0.9% saline, and cut into five transverse slices of equal thickness (2.0 mm) from the apex to the base. The slices were incubated for 10 min in phosphate-buffered 1% 2,3,5-triphenyltetrazolium chloride (TTC) at 37 °C and then fixed with 10% formalin solution. The area of infarction was stained with TTC. The infarct size were assessed by a blinded observer using Image Pro Plus.

### Masson staining

Mouse euthanasia occurred 28 days after myocardial infarction. Fresh heart samples underwent fixation with 4% (w/v) paraformaldehyde, followed by paraffin embedding and sectioning at 2 μm. Slides were stained with the Masson trichrome staining method using standard procedures. The slides were visualized using a Pannoramic MIDI Scanner (3DHISTECH Ltd., Budapest, Hungary). The staining size was assessed by a blinded observer using Image Pro Plus.

### Statistical analysis

All data are expressed as mean ± standard deviation (SD). Three independent experiments were performed. Statistical comparisons of samples were performed by Student’s *t* test for comparing two groups or one-way ANOVA followed by the Student–Newman–Keuls (SNK) post-hoc test for multiple comparisons. Grouped comparisons were carried out by two-way ANOVA. The difference with *P* < 0.05 between the groups was considered significant. All statistical analyses were performed using Prism 8.0 (GraphPad Software).

## Supplementary information

Supplementary Figure legends

supplementary figure 1

supplementary figure 2

supplementary figure 3

## Data Availability

All the data and material supporting the conclusions were included in the main paper.
