# Supplementary material for: Insulin-like growth factor binding protein-1 regulates HIF-1α degradation to inhibit apoptosis in hypoxic cardiomyocytes
Source: Cell Death Discov. 2021 Sep 16;7:242. doi: 10.1038/s41420-021-00629-3 (PMC8445926; doi:10.1038/s41420-021-00629-3)
Supplement: Supplementary file 1 — Supplementary Figure legends [file 41420_2021_629_MOESM1_ESM.docx]

**Supplementary Fig.1. 2-ME protects cardiomyocytes in hypoxic condition.**

H9c2 cells and CMs were treated with hypoxia and 2-ME (100 μM) for the indicated times. **a, b** Flow cytometry analysis was performed by Annexin V-APC/7AAD staining to calculate the cell apoptosis. Flow cytometry gating of H9c2 cells and CMs were shown. Three independent experiments were performed.

**Supplementary Fig.2. Knockdown of IGFBP-1 cannot reverse cell apoptosis caused by prolonged hypoxia.** H9c2 cells and CMs were transfected with HIF-1α siRNA and then exposed to hypoxia for 9h. **a** Protein lysates were prepared to western blotting for HIF-1α, IGFBP-1 and Bax. GAPDH was served as a loading control. Quantitative data of band gray density are shown. H9c2 cells and CMs were infected with Ad-shIGFBP-1 (MOI of 10) or negative control for 48h and then exposed to hypoxia for 9h. **b** Immunoblot was carried out to assess HIF-1α, IGFBP-1, Bax and Bcl-2 protein amounts. GAPDH was served as a loading control. Quantitative data of band gray density are shown. **c** Total RNA was obtained and assessed by qRT-PCR for BNIP-3 and p53 mRNA amounts. **d** Flow cytometry analysis was performed by Annexin V-APC/7AAD staining to calculate the cell apoptosis. **e** H9c2 cells were co-transfected with the pcDNA3.1-HIF-1α plasmid and Ad-shIGFBP-1 for 48h, and exposed to hypoxia for 9h. Immunoblot and quantification was carried out to assess HIF-1α, IGFBP-1, Bax and Bcl-2 protein amounts. GAPDH was served as a loading control. Quantitative data of band gray density are shown. **f** Flow cytometry analysis was performed by Annexin V-APC/7AAD staining to calculate the cell apoptosis. Data were presented as mean±standard deviation (SD). ^%^*P* < 0.05 vs. normoxia+si-NC group; ^&^*P* < 0.05, ^&&^*P* < 0.01 vs. hypoxia+si-NC group; ^*^*P* < 0.05, ^**^*P* < 0.01 vs. CTR group; ^#^*P* < 0.05, ^##^*P* < 0.01 vs. indicating HO group; ^$^*P* < 0.05, ^$$^*P* < 0.01 vs. HO+sh-IGFBP-1 group, one-way ANOVA followed by SNK multiple comparison test performed. Three independent experiments were performed.

**Supplementary Fig.3.** **IGFBP-1 dose-dependently decreased the interaction of HIF-1α and VHL.** H9c2 cells were transfected with plasmids indicated on top of lanes and followed by IGFBP-1 IP(**a**) and VHL IP(**b**)，then western blotting for indicated proteins. Three independent experiments were performed.
