# Supplementary figures and images for: Insulin-like growth factor binding protein-1 regulates HIF-1α degradation to inhibit apoptosis in hypoxic cardiomyocytes
Source: Cell Death Discov. 2021 Sep 16;7:242. doi: 10.1038/s41420-021-00629-3 (PMC8445926; doi:10.1038/s41420-021-00629-3)

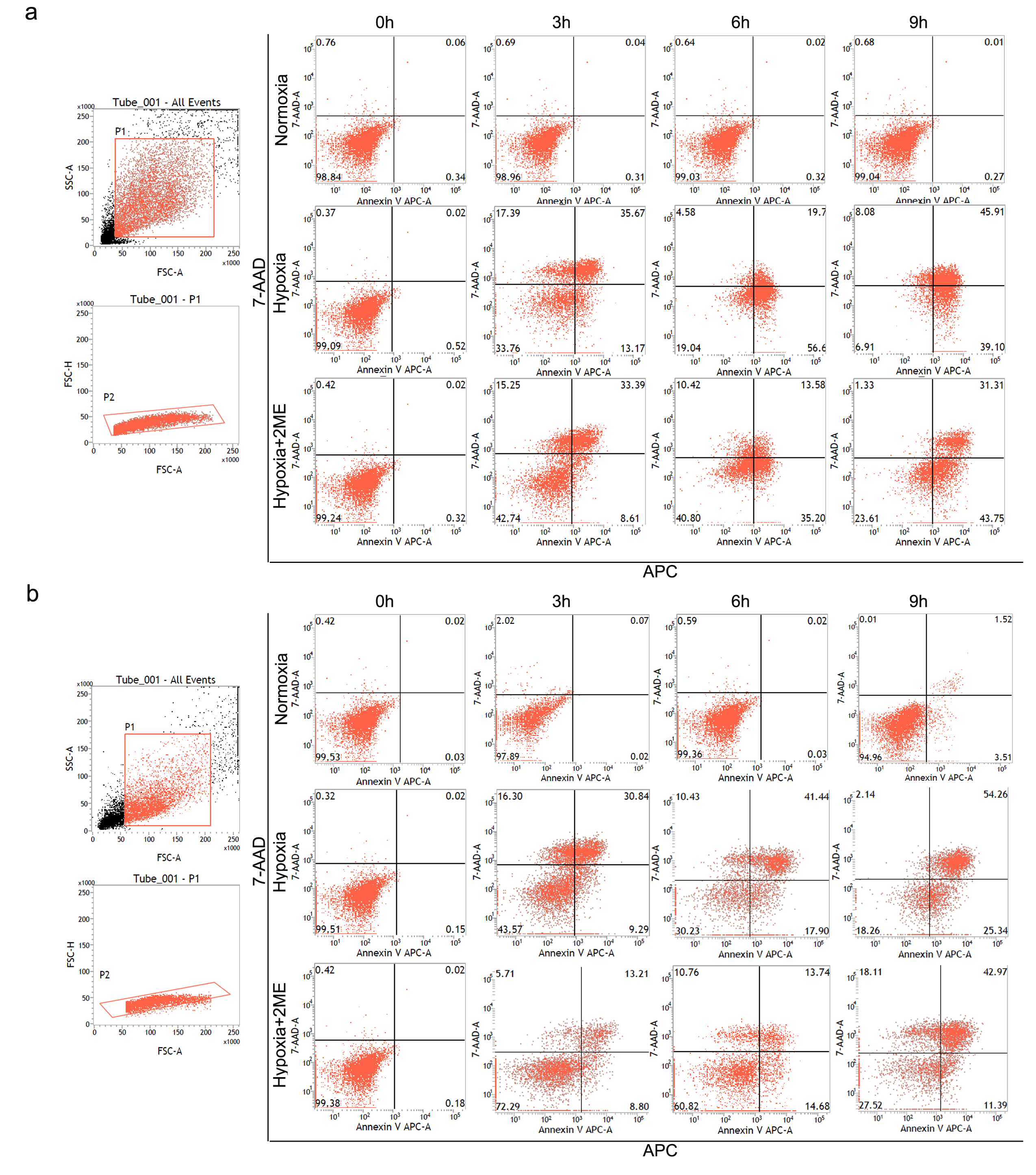

Supplement: Supplementary file 2 — supplementary figure 1 [file 41420_2021_629_MOESM2_ESM.tif]

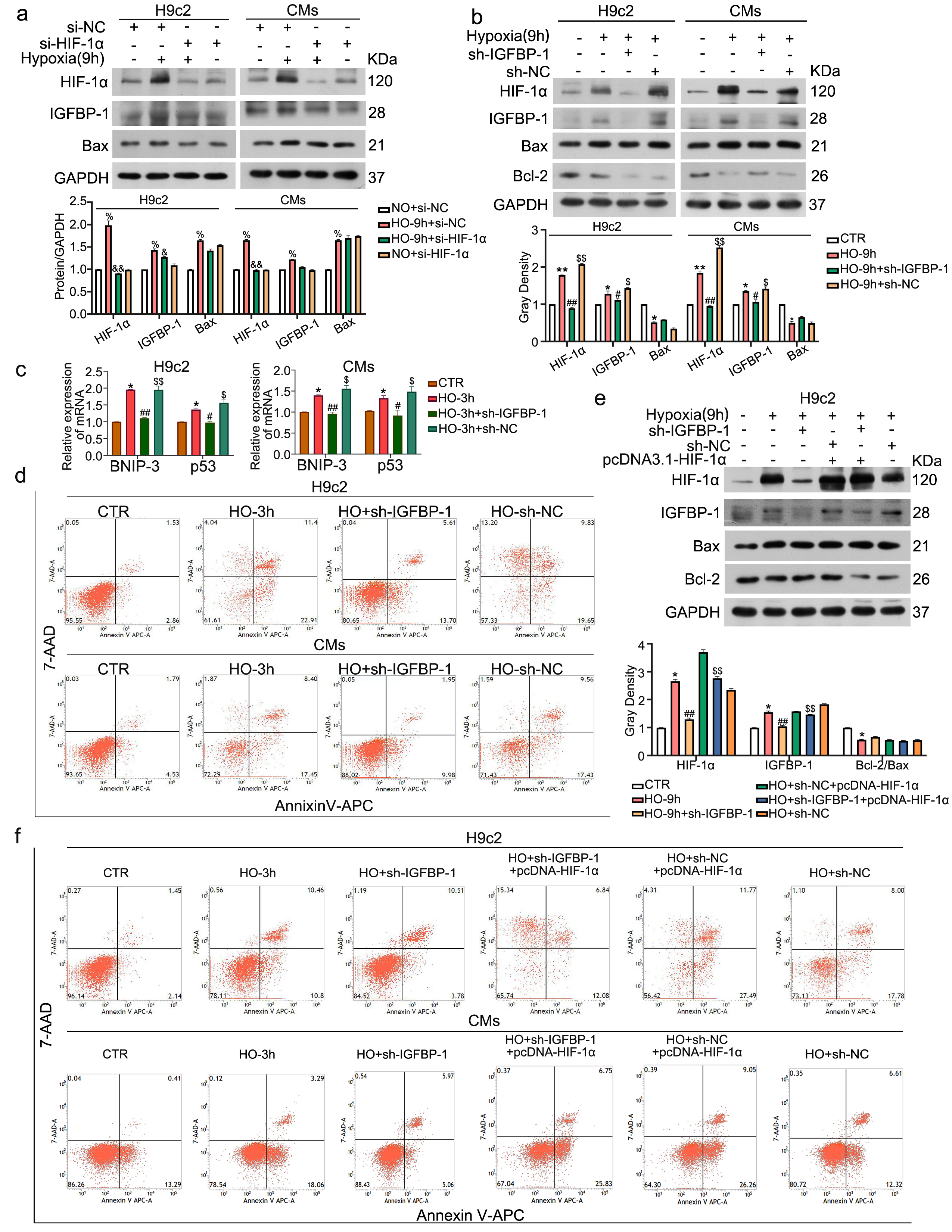

Supplement: Supplementary file 3 — supplementary figure 2 [file 41420_2021_629_MOESM3_ESM.tif]

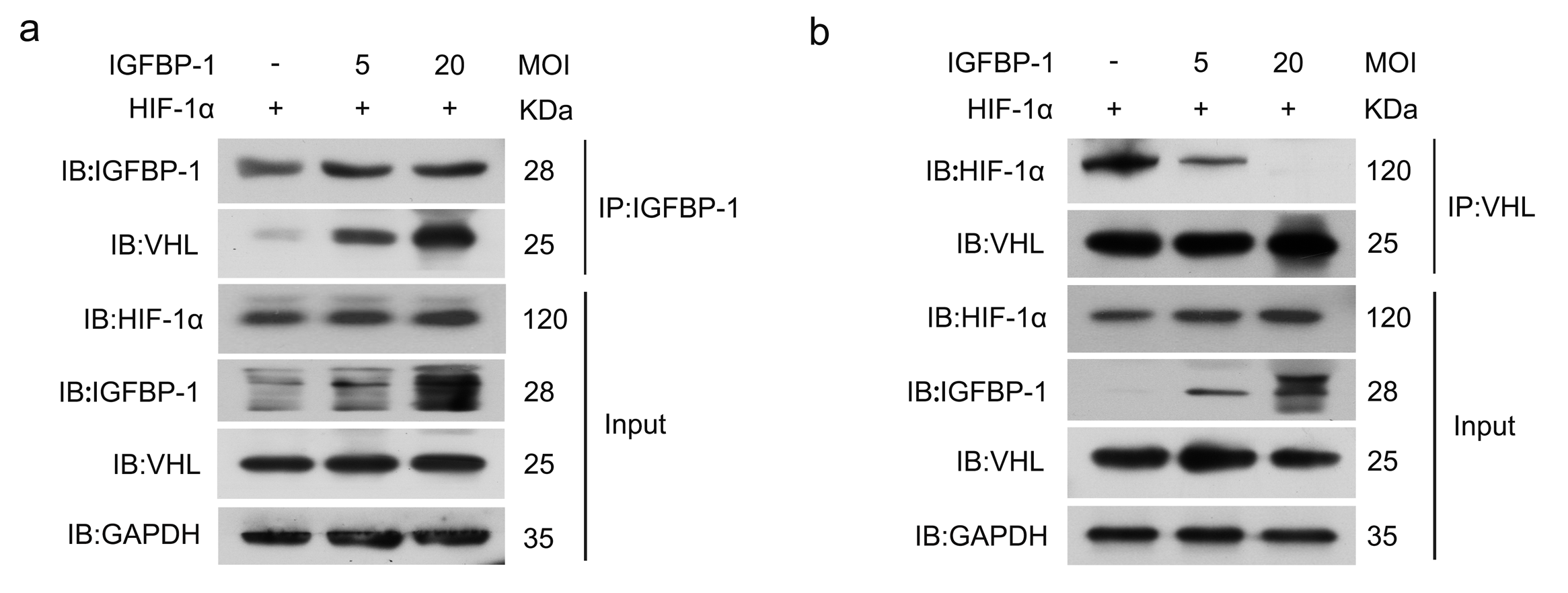

Supplement: Supplementary file 4 — supplementary figure 3 [file 41420_2021_629_MOESM4_ESM.tif]
